# Synergistic action of piperonyl butoxide with macrocyclic lactones in *Rhipicephalus microplus*

**DOI:** 10.1007/s10493-026-01164-5

**Published:** 2026-07-22

**Authors:** Larissa Claudino Ferreira, Ana Maria Santos Lima, Jordania Oliveira Silva, Thais Ferreira Feitosa, Guilherme Marcondes Klafke, Vinícius Longo Ribeiro Vilela

**Affiliations:** 1 Programa de Pós-Graduação em Ciência e Saúde Animal, Universidade Federal de Campina Grande – UFCG, Patos, PB Brazil; 2 Departamento de Medicina Veterinária, Instituto Federal da Paraíba – IFPB, Sousa, PB Brazil; 3 Instituto de Pesquisas Veterinárias Desidério Finamor, Eldorado do Sul, RS Brasil

**Keywords:** Cattle tick, Resistance, Semi-arid, Bioassay, Acaricide, Synergist, Piperonyl butoxide

## Abstract

Resistance of *Rhipicephalus microplus* to macrocyclic lactones represents a major challenge for the control of this ectoparasite, leading to substantial economic losses. The indiscriminate use of acaricides has resulted in increasingly resistant populations, highlighting the need for alternative control strategies and pharmacological approaches, such as the use of synergists to enhance acaricide efficacy against resistant ticks. This study evaluated the effect of piperonyl butoxide (PBO) as a synergist in modulating resistance to ivermectin, eprinomectin, and moxidectin in five tick populations from the semi-arid area of Northeast Brazil. Bioassays revealed widespread resistance, with variation in LC50 values among locations, likely reflecting differences in the intensity and frequency of macrocyclic lactone use and, consequently, differences in resistance intensity rather than evidence of distinct resistance mechanisms. The association of PBO with macrocyclic lactones resulted in significant synergism in the studied tick populations. A notable reduction in LC50 was observed in 3 out of 5 populations for ivermectin, 4 out of 5 populations for eprinomectin, and 3 out of 5 populations for moxidectin. Even in highly resistant populations to moxidectin, a marked decrease in LC50 was observed after PBO exposure. These results indicate a pharmacological interaction between PBO and macrocyclic lactones, consistent with a partial contribution of oxidative detoxification to the resistance phenotype. However, the absence of synergism in some populations and the persistence of high resistance levels suggest that additional mechanisms are also involved.

## Introduction

Infestation by the cattle tick *Rhipicephalus microplus* remains one of the major health challenges for cattle production in Brazil. The tropical climate favors its reproduction and dissemination (Dantas-Torres et al. 2019), contributing to the transmission of pathogens such as *Babesia bovis, Babesia bigemina,* and *Anaplasma marginale*, which directly affect cattle health and productivity (Martins et al. 2020; Alvares et al. 2026; Puentes et al. 2026), causing estimated annual losses of 3.2 billion USD (Grisi et al. 2014). Tick control relies predominantly on the application of acaricides, especially macrocyclic lactones such as ivermectin (Ferreira et al. 2023). However, the continuous and often inadequate use of acaricides has exerted strong selection pressure, and resistance has been widely documented in *R. microplus* populations from different regions of Brazil (Valsoni et al. 2020; Vilela et al. 2020; Sousa et al. 2022; Ferreira et al. 2023; Klafke et al. 2024; Heylen et al. 2024).

Macrocyclic lactones are widely used in the control of nematodes and arthropods (Putter et al. 1981). Their mechanism of action occurs mainly through binding to glutamate-gated chloride channels present in the nerve and muscle cells of invertebrates. This interaction increases chloride permeability, promotes membrane hyperpolarization, impairs nerve impulse transmission, and results in parasite paralysis and death. In addition, these molecules may also act, to a lesser extent, on channels related to gamma-aminobutyric acid (GABA), thereby reinforcing their antiparasitic effect (Strong 1987).

The main mechanisms involved in tick resistance include target-site insensitivity and enhanced metabolic detoxification (Shakya et al. 2022). Target-site insensitivity mostly involves structural alterations in the molecular targets, preventing the effective action of chemicals on the parasite’s nervous system. Resistance mechanisms may involve detoxification enzymes such as esterases, cytochrome P450 monooxygenases, and glutathione S-transferases, as well as ABC transporters, which are associated with efflux and excretion processes that reduce intracellular acaricide accumulation and may compromise control efficacy. In resistant populations, metabolic resistance may result from increased production of these enzymes or from functional alterations that enhance their catalytic efficiency, promoting faster drug metabolism and elimination, lowering its concentration in the parasite, and compromising treatment efficacy (Hemingway et al. 2004; Obaid et al. 2022).

The development of synergized formulations has been widely explored as a strategy to enhance the toxicity of pesticides against arthropods, with piperonyl butoxide (PBO) being one of the most commonly used synergists targeting oxidative detoxification pathways. PBO inhibits the catalytic activity of cytochrome P450 monooxygenases (Li et al. 2010), thereby reducing the metabolic inactivation of xenobiotics. Although cytochrome P450-mediated metabolism of macrocyclic lactones is relatively limited in cattle, these enzymes may be more relevant in ticks, particularly in the context of detoxification-associated resistance. Although historically incorporated into pyrethroid-based formulations, where it substantially increases insecticidal efficacy and restores susceptibility in resistant populations (Bingham et al. 2011), its mode of action has also prompted investigations into its potential to modify the activity of macrocyclic lactones such as ivermectin. Overexpression of cytochrome P450 genes has been associated with resistance to abamectin and ivermectin in several arthropods, and inhibition of these enzymes has been shown to increase toxicity in resistant populations (Pu et al. 2010; Xu et al. 2021). In addition, transcriptomic studies indicate that ABC transporters, including P-glycoproteins, also participate in macrocyclic lactone resistance, although current evidence suggests that PBO does not directly inhibit these transporters and that any effect on efflux mechanisms is likely indirect and limited (Pohl et al. 2012; Lespine et al. 2012; Ménez et al. 2012).

This study aimed to investigate the effect of piperonyl butoxide (PBO) as a synergist of macrocyclic lactones (ivermectin, eprinomectin, and moxidectin) in resistant *R. microplus* populations using the larval immersion test (LIT) as a tool to assess its ability to enhance acaricide activity and help characterize underlying resistance mechanisms.

## Materials and methods

### Ticks

**Fig. 1:**
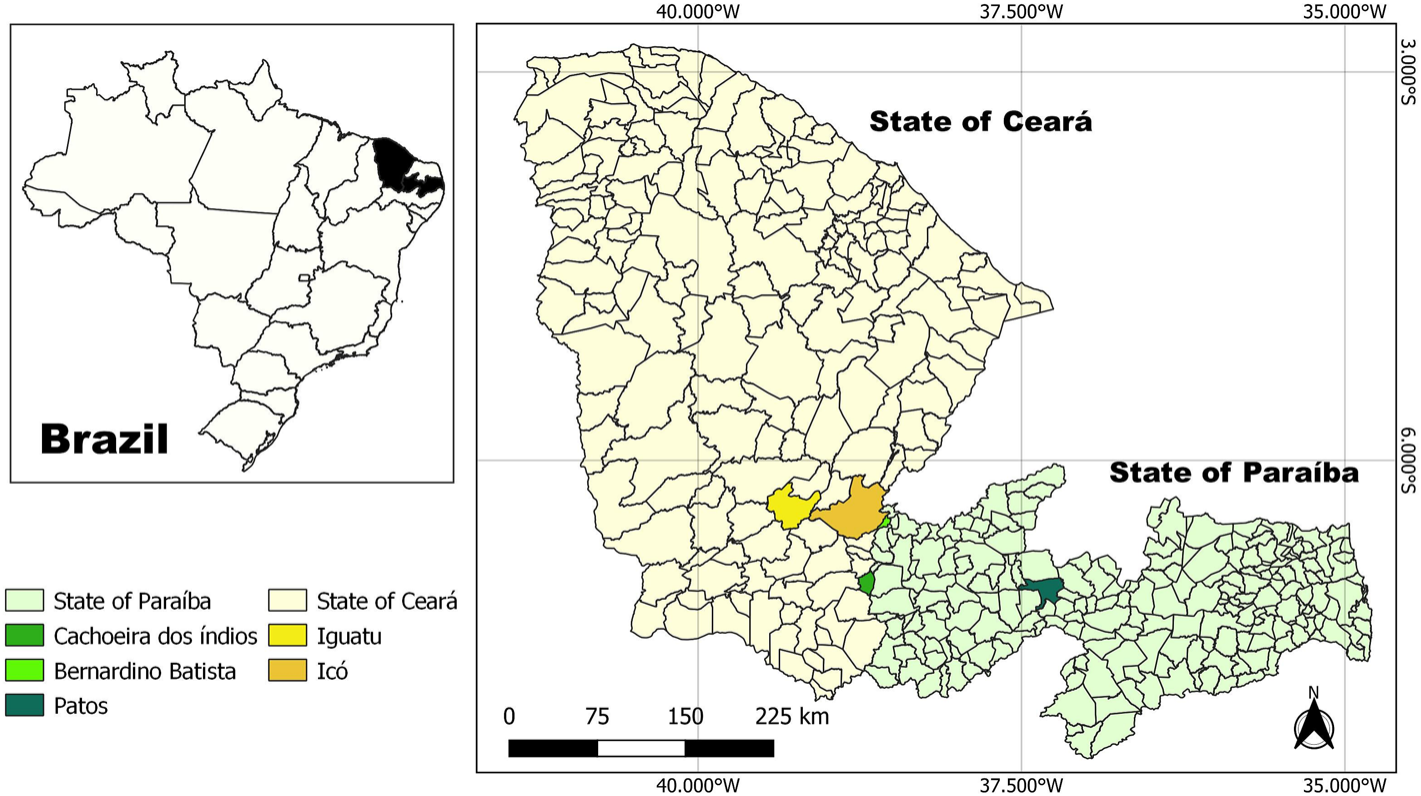
Geographic location of cattle farms from Paraíba and Ceará states used for field population sampling

The *R. microplus* Porto Alegre (POA) strain was used as the susceptible reference. Since its isolation in 1994 (Canal et al. 1995), this strain has been maintained under laboratory conditions without exposure to any acaricides. Field populations of ticks were collected in five cattle farms located in the semiarid regions of the states of Paraíba and Ceará, Northeastern Brazil. The collection sites were georeferenced (coordinates in decimal degrees, WGS84) in the municipalities of Cachoeira dos Índios (-6.945790, -38.708305), Patos (-6.992492, -37.336790), and Bernardino Batista (-6.460154, -38.556607), State of Paraíba, and Icó (-6.392776, -39.078811), and Iguatú (-6.445231, -39.382737), State of Ceará (Fig. 1). The farms were selected based on their long-term history of ivermectin and/or other macrocyclic lactone use for the control of ticks, flies, myiasis, and helminth infections.

### Tick processing

From each farm, approximately 100 engorged females were manually collected from naturally infested cattle that had not received any topical acaricide treatment for at least 30 days or injectable formulations for at least 45 days, in order to avoid treatment interference. The engorged females were placed in plastic containers (105 mm × 80 mm) with perforated lids to allow aeration and transported inside insulated boxes maintained at approximately 28 °C until arrival at the laboratory.

Female ticks from both field populations and the POA strain were handled according to FAO (2004) recommendations. The engorged females were washed with tap water, dried on paper towels, and identified to species level using morphological keys (Monteiro 2017), based on gnathosoma, coxae II–III, and spiracular plate characteristics. After identification, they were placed in plastic Petri dishes (90 mm × 22 mm) and incubated in biological oxygen demand (BOD) chambers at 27–28 °C, 85–90% relative humidity, in the dark. Females were kept for two weeks to allow oviposition. Egg masses (approximately 250 mg aliquots) were transferred to 15 mL glass vials sealed with cotton plugs to ensure air and moisture exchange. Eggs were incubated under the same controlled conditions until hatching. Larvae with 14 to 21 days after hatching were used in the bioassays.

### Chemicals

Technical-grade ivermectin (purity: 94%; batch number: BCCG6701), eprinomectin (purity: 98%; batch number: LRAC6950), and moxidectin (purity: 98%; batch number: BCCB5409) were obtained from Sigma Chemical Co. (St. Louis, MO, USA). Piperonyl butoxide (PBO; purity: 95%; batch number: FDTC8761 Sigma–Aldrich Chemical Co., St. Louis, MO, USA) was used as a synergist to inhibit cytochrome P450 oxidase enzymes (Pohl et al. 2012; Le Gall et al. 2018). PBO was tested at a final concentration of 0.0025%, selected based on previously published experimental conditions for synergism assays in *R. microplus* (Le Gall et al. 2018). The present study did not assess the dose–response relationship of PBO.

### Larval immersion test (LIT)

Bioassays were conducted using the Larval Immersion Test (LIT), as described by Klafke et al. (2012) and modified by Ferreira et al. (2023). Acaricides were diluted in a 2% Triton X-100 solution in absolute ethanol (ETH-TX2%) for eprinomectin, and in 2% Triton X-100 with pure acetone (ACTX-2%) for ivermectin and moxidectin. Control treatments used the same diluent without acaricide. Technical-grade active ingredients (AI) were first dissolved at 1% in 10 mL of the corresponding solvent to prepare stock solutions. For each test, 100 µL of the stock solution were added to 9.9 mL of distilled water, yielding a mixture containing 0.01% AI, 1% acetone or ethanol, and 0.02% Triton X-100. This solution was serially diluted in a mixture of 1% acetone or ethanol and 0.02% Triton X-100 to obtain immersion solutions providing a larval mortality gradient between 1 and 99%. Final test concentrations ranged from 5 to 2000 ppm. For each concentration, 500 µL of the respective test solution were used as the immersion volume in each of three 1.5 mL microtubes (Axygen, Union City, CA, USA). Approximately 100 larvae were added to each tube using a fine brush. Tubes were capped and vigorously shaken to ensure full contact between larvae and solution. After 10 min of immersion, larvae were removed, dried on paper towels, and placed on folded filter papers sealed with metal clips on three sides. The packets were incubated in BOD chambers in the dark at 27–28 °C and 85–90% relative humidity for 24 h. Mortality was then assessed by counting live and dead larvae. Larvae that were motionless or only exhibited leg twitching without locomotion were considered dead. Tests were performed in triplicates.

### LIT with cytochrome P450 oxidase inhibitor

Piperonyl butoxide (PBO) was used to inhibit cytochrome P450 oxidase activity, aiming to evaluate its effect on detoxification mechanisms in *R. microplus* larvae exposed to macrocyclic lactones. PBO was tested in combination with ivermectin, eprinomectin, and moxidectin at a final concentration of 0.0025%, according to Le Gall et al. (2020), prepared in 5 mL of a 1% acetone + 0.02% Triton X-100 solution. Larvae were exposed using the same LIT procedure described above. For the “control + PBO” treatment, the diluent contained only the synergist without acaricide.

### Statistical analysis

Probit analysis was performed on bioassay data using PoloPlus software (LeOra Software 2003). For each test, the median lethal concentration (LC50), 95% confidence intervals (CI), and slope of the regression line were estimated. Resistance factors (RF) were calculated as the ratio of the LC50 of the field population to that of the susceptible reference strain (POA). The synergism factor (SF) was obtained by dividing the LC50 of the acaricide alone by the LC50 of the acaricide + PBO combination (Robertson et al. 2007; Le Gall et al. 2018).

The presence or absence of a significant difference between treatments with and without PBO within each population was determined by comparing the overlap of the 95% confidence intervals. In vitro resistance of field populations was assessed separately by calculating the resistance factor (RF), defined as the ratio between the LC50 of each field population and that of the susceptible reference strain (POA).

## Results

All five farms managed cattle under semi-intensive production systems, with native Caatinga pastures interspersed with cultivated grasses (*Panicum maximum*, *Pennisetum purpureum*, and *Cenchrus ciliaris*). Herd sizes ranged from 23 to 80 animals (average of 40), consisting mainly of zebuine and taurine dairy breeds. All farms reported long-term use of injectable ivermectin (1%) and other acaricide classes, including pyrethroids, organophosphates, and formamidines, applied by spraying. In Bernardino Batista (PB) and Icó (CE), acaricides were applied weekly throughout the year, whereas in Cachoeira dos Índios (PB), Iguatu (CE), and Patos (PB), treatments were carried out every 8 days during the rainy season (January–May) and every 15–30 days during the dry season (June–December), or whenever engorged females were observed on animals. The main target pests were *R. microplus* and *Haematobia irritans*. In Cachoeira dos Índios, Bernardino Batista, and Icó, tick infestations were reported as the main health problem in the herds, whereas in Patos and Iguatu, bovine babesiosis and anaplasmosis were more frequently mentioned. Overall, these management characteristics indicate intense and repeated exposure of tick populations to acaricides, which may have contributed to the selection pressure associated with the high resistance levels observed in the bioassays.

All *R. microplus* populations tested showed resistance to ivermectin, eprinomectin, and moxidectin, with high resistance factors (RF) indicating widespread and intense resistance. Among the three macrocyclic lactones, moxidectin generally showed the highest RF values, followed by eprinomectin and ivermectin.

For ivermectin (Table 1), RF values ranged from 12.26 (Iguatu-CE) to 78.68 (Patos-PB). The addition of PBO significantly reduced the LC50 values in three populations, Icó-CE (SF = 3.41), Patos-PB (SF = 2.98) and Cachoeira dos Índios (SF = 1.76). For eprinomectin (Table 2), all populations exhibited high resistance (RF = 114.8–667.3). The addition of PBO significantly reduced the LC50 values in four populations, Cachoeira dos Índios-PB (SF = 6.96), Icó-CE (SF = 3.81), Iguatú-CE (SF = 2.45), and Patos-PB (SF = 2.34). However, the LC50 estimate for the Bernardino Batista population should be interpreted with caution due to the very wide 95% confidence interval, which indicates low precision for this specific estimate. Moxidectin (Table 3) presented the highest overall resistance levels (RF = 143.1–760.1). In the presence of PBO, LC50 values were significantly reduced in three populations, Bernardino Batista-PB (SF = 4.32), Iguatú-CE (SF = 4.02) and Icó-CE (SF = 1.69).

**Table 1:** Comparative efficacy of ivermectin alone and in combination with piperonyl butoxide on *Rhipicephalus microplus* larvae

Population / location	Treatment	n	Slope (SE)	LC50 (95% CI, ppm)	RF	SF
POA (susceptible strain)	IVM	1987	1.610 (0.073)	25.2 (16.6–36.1)	–	–
Cachoeira dos Índios – PB	IVM	3395	2.688 (0.095)	607.8 (509.0–718.9)	24.09	–
	IVM + PBO	3503	1.864 (0.057)	345.2 (275.9–423.7)*	–	1.76
Patos – PB	IVM	1863	2.165 (0.180)	1984.9 (1641.5–2543.5)	78.68	–
	IVM + PBO	1688	1.977 (0.107)	666.5 (531.6–836.3)*	–	2.98
Bernardino Batista – PB	IVM	2099	2.450 (0.152)	1130.0 (900.7–1452.4)	44.81	–
	IVM + PBO	2213	2.508 (0.126)	985.1 (827.2–1174.4)	–	1.15
Icó – CE	IVM	2214	3.330 (0.161)	483.0 (403.9–567.1)	19.15	–
	IVM + PBO	2271	6.142 (0.326)	141.8 (132.7–151.2)*	–	3.41
Iguatu – CE	IVM	2175	2.656 (0.118)	309.1 (267.7–353.4)	12.26	–
	IVM + PBO	2352	3.081 (0.144)	226.5 (181.5–277.7)	–	1.36

n = number of larvae tested; IVM = ivermectin; PBO = piperonyl butoxide; SE = Standard error; LC50 = lethal concentration (ppm of active ingredient); RF = resistance factor: tested population LC50/ susceptible strain; SF = synergism factor: LC50 IVM/LC50 IVM + PBO; * indicates LC50 significantly lower in the presence of PBO

**Table 2:** Comparative efficacy of eprinomectin alone and in combination with with piperonyl butoxide on *Rhipicephalus microplus* larvae

Population / location	Treatment	n	Slope (± SE)	LC50 (95% CI, ppm)	RF	SF
POA (susceptible strain)	EPRI	1906	1.340 (0.054)	32.4 (9.7–76.9)	–	–
Cachoeira dos Índios – PB	EPRI	4060	1.120 (0.197)	21,657.6 (7600.9–80,090.9)	667.3	–
	EPRI + PBO	3622	1.485 (0.106)	3110.1 (2396.9–4747.5)*	–	6.96
Patos – PB	EPRI	2027	1.773 (0.254)	6455.3 (4356.9–12,986.6)	198.9	–
	EPRI + PBO	2027	2.547 (0.275)	2759.2 (2381.1–3389.6)*	–	2.34
Bernardino Batista – PB	EPRI	1977	1.740 (0.312)	7294.9 (4009.0–52998.6)	224.8	–
	EPRI + PBO	1826	2.713 (0.423)	3063.1 (2343.1–6625.7)	–	2.38
Icó – CE	EPRI	2591	0.853 (0.059)	4544.6 (2658.5–10,771.7)	140.0	–
	EPRI + PBO	1969	1.363 (0.094)	1192.8 (985.1–1478.0)*	–	3.81
Iguatu – CE	EPRI	2395	0.449 (0.083)	3726.8 (2421.3–4058.5)	114.8	–
	EPRI + PBO	1901	1.579 (0.124)	1521.4 (1231.0–1972.1)*	–	2.45

n = number of larvae tested; EPRI = eprinomectin; PBO = piperonyl butoxide; SE = Standard error; LC50 = lethal concentration (ppm of active ingredient); RF = resistance factor: tested population LC50/ susceptible strain; SF = synergism factor: LC50 EPRI/LC50 EPRI + PBO; * indicates LC50 significantly lower in the presence of PBO

**Table 3:** Comparative efficacy of moxidectin alone and in combination with with piperonyl butoxide on *Rhipicephalus microplus* larvae

Population / location	Treatment	n	Slope (± SE)	LC50 (95% CI, ppm)	RF	SF
POA (susceptible strain)	MOX	2688	5.940 (0.368)	0.298 (0.283–0.314)	–	–
Cachoeira dos Índios – PB	MOX	1198	3.523 (0.186)	42.6 (35.6–49.8)	143.1	–
	MOX + PBO	1254	7.274 (0.377)	49.2 (45.9–52.6)	–	1.15
Patos – PB	MOX	1669	6.309 (0.650)	84.1 (78.2–90.9)	282.4	–
	MOX + PBO	1767	3.144 (0.137)	87.4 (67.7–112.5)	–	0.96
Bernardino Batista – PB	MOX	2099	2.450 (0.152)	101.9 (87.3–116.6)	342.3	–
	MOX + PBO	1684	6.615 (0.712)	23.5 (17.4–29.6)*	–	4.32
Icó – CE	MOX	2251	2.915 (0.128)	65.9 (57.2–76.1)	221.3	–
	MOX + PBO	2220	8.559 (0.814)	39.1 (37.0–41.7)*	–	1.69
Iguatu – CE	MOX	2353	4.745 (0.453)	167.6 (102.3– 216.2)	562.4	–
	MOX + PBO	1767	6.573 (0.345)	56.4 (42.0–74.1)*	–	2.97

n = number of larvae tested; MOX = moxidectin; PBO = piperonyl butoxide; SE = Standard error; LC50 = lethal concentration (ppm of active ingredient); RF = resistance factor: tested population LC50/ susceptible strain; SF = synergism factor: LC50 MOX/LC50 MOX + PBO; * indicates LC50 significantly lower in the presence of PBO

**Fig. 2:**
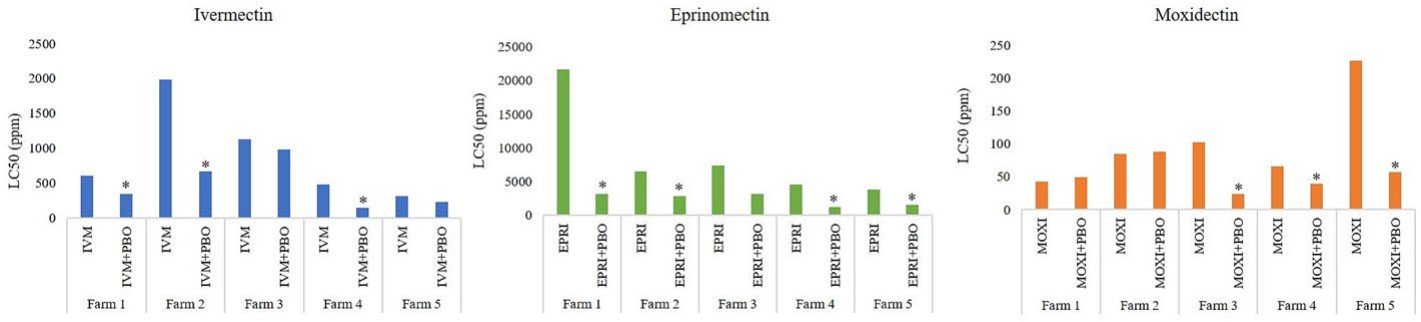
Graphical comparison of LC50 values for ivermectin, eprinomectin, and moxidectin tested alone or in combination with piperonyl butoxide against *R. microplus* larvae from five field populations. Farm 1 = Cachoeira dos Índios – PB; Farm 2 = Patos – PB; Farm 3 = Bernardino Batista – PB; Farm 4 = Icó – CE; Farm 5 = Iguatú – CE. Bars represent LC50 values (ppm of active ingredient). IVM = ivermectin; EPR = eprinomectin; MOXI = moxidectin; PBO = piperonyl butoxide. * indicates LC50 significantly lower in the presence of PBO

The graphical comparisons of the LC50 values for the three compounds alone or in the presence of PBO are shown in Fig. 2. Overall, the inclusion of PBO consistently lowered LC50 in most populations and increased the apparent toxicity of the macrocyclic lactones, although the reduction in resistance intensity was partial and variable among populations and compounds.

## Discussion

All field populations of *R. microplus* evaluated in this study exhibited resistance to ivermectin, eprinomectin, and moxidectin, confirming the widespread nature of macrocyclic lactone (ML) resistance in this area of northeastern Brazil (Vilela et al. 2020; Ferreira et al. 2022; Alvares et al. 2025). The high resistance factors recorded reflect the long history of intensive and often indiscriminate use of MLs in the analyzed cattle farms, primarily for the simultaneous control of ticks, flies, and gastrointestinal nematodes. Similar findings have been reported in other parts of Brazil (Klafke et al. 2012) and abroad (Nazim et al. 2022), indicating that resistance to this chemical group has become a major constraint to effective tick control.

Although ivermectin was the only ML formulation reportedly used on the surveyed farms, all populations also showed resistance to eprinomectin and moxidectin, indicating cross-resistance among these compounds, as previously reported (Ferreira et al. 2023; Becker et al. 2025). The frequent use of injectable ivermectin for ecto- and endoparasite control may have contributed to selection pressure on *R. microplus* populations (Lifschitz et al. 2024; Vilela et al. 2020). Due to their structural similarity and shared mode of action, side-resistance between ivermectin and eprinomectin is expected, as both belong to the avermectin subgroup and are derivatives of avermectin B1 (Ménez et al. 2016). However, despite these similarities, moxidectin has distinct pharmacokinetic characteristics, including greater plasma and tissue persistence. As a milbemycin, moxidectin may retain activity even in the presence of avermectin resistance (Bourguinat et al. 2011), and resistance to this compound is often reported to develop more slowly than resistance to ivermectin. In this context, the resistance to moxidectin observed in the present study may be consistent with cross-resistance under shared selection pressure, although specific studies would be necessary to confirm this relationship.

The lower LC50 observed for moxidectin in the susceptible strain (POA), compared with ivermectin and eprinomectin, may reflect intrinsic physicochemical and pharmacodynamic differences among macrocyclic lactones. Unlike ivermectin and eprinomectin, which belong to the avermectin group, moxidectin is a milbemycin and lacks the disaccharide substituent present in avermectins, a structural difference that may influence its lipophilicity, penetration, and interaction with target sites in tick larvae. Under the conditions of the present bioassay, these characteristics may have contributed to the greater intrinsic toxicity of moxidectin in the susceptible population. However, this finding should be interpreted cautiously, as it reflects the response of a laboratory-maintained strain under in vitro conditions and does not necessarily indicate greater field efficacy.

The association between PBO and macrocyclic lactones resulted in synergism in 66.6% (10/15) of the assays; however, the magnitude of this effect was only partial, and resistance factors remained extremely high even in the presence of the synergist. Although reductions in LC50 and increases in synergism factor were observed in several populations, the residual resistance levels remained high in most cases, indicating that the biological effect of PBO was limited to a partial restoration of susceptibility rather than a complete recovery of acaricide efficacy.

This pattern is consistent with a partial contribution of oxidative detoxification to the observed response, although it does not allow direct confirmation of cytochrome P450 involvement as a specific resistance mechanism (Rouck et al. 2023). Similar observations were reported in studies using cyclosporin A, in which partial reductions in LC50 suggested a complementary rather than dominant role of ABC transporters such as P-glycoproteins, whose involvement in macrocyclic lactone resistance has been previously documented (Feyereisen et al. 2015; Ferreira et al. 2022). Combined with evidence from the literature showing variable patterns of P450 overexpression among arthropods resistant to abamectin and other macrocyclic lactones (Pu et al. 2010; Xu et al. 2021), these findings support a multifactorial and complex resistance profile in which PBO inhibits only a subset of the metabolic pathways involved. Thus, the synergistic effect observed reflects a limited contribution of oxidative metabolism, consistent with a polygenic resistance scenario in which multiple detoxification and efflux processes coexist. In the ivermectin experiments, only three of the five tested populations showed synergism.

Since all assays were conducted using larvae obtained from the same herds and under identical management conditions, the variability observed in synergism is more likely explained by intrinsic molecular differences among the populations such as variation in the expression or activity of detoxification enzymes and different contributions of resistance mechanisms or differential reliance on multifactorial resistance mechanisms, including P-glycoprotein–mediated efflux through the overexpression of ABC transporters rather than by external or environmental factors. Although PBO can act on this type of resistance, its effectiveness is lower than that observed against P450-mediated mechanisms. Other possible contributing factors include alterations in cuticular barriers, target-site mutations, and the activity of esterases and glutathione S-transferases (Shakya et al. 2022; Furnival-Adams et al. 2024). These factors may reduce the impact of oxidative metabolism inhibition by PBO, helping to explain why, in some populations, the inhibition of P450 enzymes, with possible indirect effects on ABC transporters, was sufficient to reveal synergism, whereas in others, resistance may persist through additional mechanisms (Rouck et al. 2023).

Thus, the response patterns observed for eprinomectin and moxidectin may indicate a greater contribution of oxidative detoxification pathways in some populations, whereas the lower and more variable response to PBO in ivermectin assays suggests a more complex resistance profile. However, because no molecular or biochemical assays were performed, these interpretations should be considered cautiously and regarded as indirect evidence based on phenotypic bioassays.

A limitation of this study is that the susceptible reference strain (POA) was not tested in the presence of PBO. Therefore, it was not possible to distinguish baseline pharmacological potentiation from resistance-specific metabolic inhibition. For this reason, the observed LC50 shifts should be interpreted cautiously as evidence of pharmacological interaction, rather than direct confirmation of a specific resistance mechanism. In some assays, the addition of PBO was associated with steeper slopes, indicating a more homogeneous concentration–response pattern. Although this may reflect increased uniformity of larval response under synergist exposure, the absence of complementary assays prevents distinction between gradual pharmacological modulation and possible acute toxic interference. Therefore, these slope changes should be interpreted cautiously. Another limitation of the eprinomectin dataset was the low precision of the LC50 estimate for the Bernardino Batista population, as indicated by its very wide 95% confidence interval. Therefore, this specific result should be interpreted cautiously.

The PBO concentration used in this study was selected for experimental purposes under in vitro conditions and should not be directly interpreted as representative of in vivo use. Therefore, although the results demonstrate pharmacological interaction in the bioassay, the formulation feasibility, safety, stability, residue implications, and practical applicability of this combination in cattle production systems remain to be established.

## Conclusion

The association of piperonyl butoxide with macrocyclic lactones produced a synergistic effect in most assays, indicating a pharmacological interaction consistent with a partial contribution of oxidative detoxification to the resistant phenotype. However, the incomplete reduction in LC50 values and the persistence of high resistance levels suggest that macrocyclic lactone resistance in these *R. microplus* populations is multifactorial and may involve additional mechanisms, including efflux transporters, target-site alterations, cuticular barriers, and other detoxification pathways. Therefore, PBO should be interpreted as a useful tool to investigate oxidative detoxification, but not as evidence of a single or predominant resistance mechanism.

## Data Availability

We declare all data is being provided within this manuscript.
